# Spectra library assisted de novo peptide sequencing for HCD and ETD spectra pairs

**DOI:** 10.1186/s12859-016-1330-0

**Published:** 2016-12-23

**Authors:** Yan Yan, Kaizhong Zhang

**Affiliations:** Department of Cumputer Science, Faculty of Science, University of Western Ontario, London, Canada

**Keywords:** De novo peptide sequencing, Spectra library, Higher-energy collisional dissociation, Electron transfer dissociation

## Abstract

**Background:**

De novo peptide sequencing via tandem mass spectrometry (MS/MS) has been developed rapidly in recent years. With the use of spectra pairs from the same peptide under different fragmentation modes, performance of de novo sequencing is greatly improved. Currently, with large amount of spectra sequenced everyday, spectra libraries containing tens of thousands of annotated experimental MS/MS spectra become available. These libraries provide information of the spectra properties, thus have the potential to be used with de novo sequencing to improve its performance.

**Results:**

In this study, an improved de novo sequencing method assisted with spectra library is proposed. It uses spectra libraries as training datasets and introduces significant scores of the features used in our previous de novo sequencing method for HCD and ETD spectra pairs. Two pairs of HCD and ETD spectral datasets were used to test the performance of the proposed method and our previous method. The results show that this proposed method achieves better sequencing accuracy with higher ranked correct sequences and less computational time.

**Conclusions:**

This paper proposed an advanced de novo sequencing method for HCD and ETD spectra pair and used information from spectra libraries and significant improved previous similar methods.

## Background

Tandem mass spectrometry (MS/MS) is a dominant technique nowadays for peptide sequencing [1]. A typical MS/MS experiment usually includes the following steps: protein mixtures are first digested into suitably sized peptides, and then the peptides are ionized via an ionization process. After that, selected peptides (also named as precursor ions) are further broken into fragment ions using different fragmentation techniques, and their tandem mass spectra (MS/MS spectra) are output [2]. MS/MS spectra usually contain two kinds of information of each ion detected, the mass-to-charge (*m*/*z*) value and the intensity.

In MS/MS experiments, precursor ions are broken into various kinds of fragment ions, among which, the commonly observed ones are named *a*-, *b*-, *c*-, *x*-, *y*-, and *z*-ions according to the cleavage sites on the peptide backbones. Different fragmentation techniques used in MS/MS yield differing dominant types of fragment ions. Collision-induced dissociation (CID) and higher-energy collisional dissociation (HCD) yield *b*-ions and *y*-ions as dominating ions. Electron capture dissociation (ECD) and electron transfer dissociation (ETD) preferentially produce variants of *c*-ions and *z*-ions, and occasionally *a*-ions [3–5].

Different kinds of computational methods including database search, de novo sequencing, and spectra library search have been developed for peptide sequencing using various MS/MS data. In database search, theoretical peptide spectra are computed from an existing protein database and peptides are identified by matching the theoretical spectra to experimental spectra. Spectra library search is a relatively new method that uses pre-build spectra libraries instead of protein database as the reference for matching. Spectra libraries contain annotated experimental spectra. This kind of method is claimed to be superior in speed and sensitivity, compared to the database search [6]. The limitation of these two kinds of methods is obvious that they can only identify peptides that are included in protein database or spectra libraries. De novo sequencing, on the other hand, interprets spectra directly using the masses of amino acids [7–10]. No prior database nor library is needed. Therefore, this kind of method has the potential to identify peptides that are not included in protein database and spectra libraries. The development of de novo sequencing used to be limited by insufficient information from an MS/MS spectrum itself, especially when the spectrum quality is low. However, with the recent development of high mass-accuracy MS/MS, alternative fragmentation techniques, and the idea of using multiple spectra from the same peptide for sequencing [11, 12], de novo sequencing has shown promising developments[3, 13–17]. Therefore, this study focuses on de novo peptide sequencing methods.

A recent popular way of peptide sequencing is to combine different kinds of methods properly in order to achieve superior performance, for example, tag based searching methods [18, 19] can be viewed as a combination of de novo sequencing and database search. This kind of methods usually first produce partial sequences using de novo sequencing, called tags, from an MS/MS spectrum, and then use these tags to search against a protein database. The use of tags can dramatically reduce the search space and time needed.

Nowadays, with large amount of MS/MS spectra produced and sequenced, spectra libraries are expending. Information extracted from these experimental spectra in libraries can be used to enhance de novo sequencing performance. Previously, we have produced a series of de novo sequencing methods for alternative spectra including HCD and ECD/ETD spectra, and for multiple spectra generated by the same peptide [9–11]. We believe that information extracted from spectra libraries could help with these existing de novo sequencing methods. In this study, we use spectra libraries as training datasets to improve our previously proposed method for HCD and ETD spectra pairs.

Our previously proposed method for HCD and ETD spectra pairs [11] is based on the widely used spectrum graph model with proper modifications. In this method, a pair of spectra are first merged into one spectrum (the detailed merging steps are introduced in the following section), and a new spectrum graph with multiple types of edges is built on the merged spectrum. Then, the method uses peptide tags to separate the whole sequencing into small regions, and integrates amino acid composition (AAC) information into the graph model. Partial candidate sequences inferred from the graph are assembled together to be final candidates, and a ranking scheme is applied at last to find the best match. Several spectrum-specific features are applied to the graph model for sequencing. Since spectra libraries consist of annotated experimental spectra, features extracted from them are expected to reflect properties of real MS/MS spectra, and have the potential to improve our previous method. In this study, we propose an improved de novo sequencing method with the use of spectra libraries.

## Methods

### Spectra merging improvement

In our previously proposed method, in order to merge a pair of HCD and ETD spectra to be one spectrum for sequencing, peaks from both spectra satisfying certain criteria are selected. Having spectra libraries, we can evaluate these criteria and assign significant scores to them. Therefore, a new dimension of information, the significant score of each selected peak, can be added into the sequencing method. That is to say, previously we just decide to select a peak *i* into the merged spectrum (denoted as *S*_*m*_) or not; but now, each selected *i* has a score associated with it, denoted as *s* *s*_*i*_, indicating the confidence level that it is a real fragment ion rather than a noisy peak. This score can be used as additional information in the following sequencing and candidate ranking steps. Next, we introduce the peak selection criteria and significant score calculation in details.

Two major selection criteria used in the spectra merging step are amino acid mass difference and ion complementarity. For the amino acid mass difference, a peak *v* in an experimental spectrum *S* is selected if there are two peaks *u* and *t* in *S*, one of the masses is smaller than *v* and the other is larger than *v*, and both *u* and *t* have mass difference to *v* equal to one of the 20 amino acid masses. For the ion complementarity, ions *u* and *v* in an experimental spectrum are both selected if masses of *u* and *v* satisfying the complementary ion relationship. Since ions may loss small molecules and contain various charge values, these variations are considered during the selection.

In order to evaluate the selection criteria and assign significant scores, spectra libraries are used as training datasets to calculate accuracies of the criteria. To be specific, on each spectrum in a spectra library, denoted as *S* *L*_*k*_, we apply the above selection criteria and then check how many of the selected peaks are real fragment ions (accuracy of such selection). The average accuracy on all *S* *L*_*k*_ in the library is used as the significant score of such selection criterion.

To describe the selection and score calculation clearly, we denote *A* as the set of 20 amino acids, and *a*_*i*_∈*A* as a certain amino acid. *a*_*i*_ is also used to represent its residue mass. *S*_*e*_ represents an ETD spectrum and *S*_*h*_ represents a HCD spectrum. *m*_*loss*_ is defined to be the mass of some small molecules or groups lost from fragment ions, which include *H*_2_ *O* and *N* *H*_3_. *u* ^+^,*v* ^+^, and *t* ^+^ are the *m*/*z* values of *u*,*v*, and *t* in charge state +1. *θ* is a given threshold; and *m*_*p*_ is the parent peptide mass. Relationships for selection and score calculation are summarized in Table 1. In the table, $N_{ion}^{select}$ and $N_{ion}^{real}$ are the number of selected ions using the selection criteria and real fragment ions in all selected ones, respectively. $N_{comp}^{select}$ and $N_{comp}^{real}$ are the number of selected complementary ions using the selection criteria and real fragment ions in all selected ones, respectively. *a*_*i*_,*a*_*j*_∈*A*, and *σ* can be 0 or *m*_*loss*_ (considering the loss of small molecules of fragment ions).

**Table 1:** Relationships for selection and score calculation

Relationship	Ions selected	Score calculation on
		spectrum *S* *L*_*k*_
∣(*v* ^+^−*u* ^+^)−*a*_*i*_±*σ*∣≤*θ*	*v*	$S^{aa}_{k}=\frac {N_{ion}^{real}}{N_{ion}^{select}}$
and ∣(*t* ^+^−*v* ^+^)−*a*_*j*_±*σ*∣≤*θ*	(middle ion)	
∣*m*_*p*_+2*m*_*H*_−(*v* ^+^+*u* ^+^)±*σ*∣≤*θ*	*v* and *u* if *u*,*v*∈*S*_*c*_	$S^{comp}_{k}=\frac {N_{comp}^{real}}{N_{comp}^{select}}$
∣*m*_*p*_+3*m*_*H*_−(*v* ^+^+*u* ^+^)±*σ*∣≤*θ*	*v* and *u* if *u*,*v*∈*S*_*e*_	

In the above selection, *u*,*v*, and *t* in multiple charges up to *n*−1 are considered, where *n* is the precursor ion charge. In a spectrum from a charge *n* precursor ion, typically the highest charged ion is the precursor ion itself, and fragment ions are in lower charge states (from +1 to *n*−1). Since the purpose here is to select real fragment ions, ion charges up to *n*−1 is considered. Basically, *n*−1 assumptions of charge values for each ion are built during the calculation, and the *m*/*z* values in charge state +1 is used for selection.

Finally, we summarize all scores calculated from spectra libraries in Table 2. We denote the total spectra number in a library is *L*. If a peak *v* in an experimental HCD or ETD spectrum satisfies multiple selection criteria, its final score *s* *s*_*v*_ is the sum of the all scores from the selection.

**Table 2:** Scores calculated using spectra library *SL*

Feature	Score calculation
Amino acid difference	$S^{aa}_{SL}=\frac {1}{L}\sum ^{k=L}_{k=1}S^{ion}_{k}$
Ion complementarity	$S^{comp}_{SL}=\frac {1}{L}\sum ^{k=L}_{k=1}S^{comp}_{k}$

Here, we give a simple example to show the spectra merging and score assignment. We use the same example spectra as the ones in [11] with addition of significant scores. Assume the *m*/*z* values of two experimental spectra are *S*_*c*_={130,199,277,346} (represent a HCD spectrum) and *S*_*e*_={132,182,234} (represent an ETD spectrum). The parent mass is *m*_*p*_=492. The charge states of *S*_*c*_ and *S*_*e*_ are +2 and +3, respectively. The lost small molecule is *H*_2_ *O*, and *m*_*loss*_=18. Integer values are used for all masses here to simplify the calculation and focus on the method process.

In the above selection, ions in multiple charges up to *n*−1 are considered, where *n* is the precursor ion charge. Therefore, we build *n*−1 assumptions of each spectrum and convert all ions to charge state +1. For the two spectra in the example, three associated spectra are generated. A spectrum with subscripts *i* *t* *o*1(*i*=1,2) represents the spectrum with charge +1 *m*/*z* values of the ions when assuming all ions are in charge state *i*. Different fonts and underlining of values are explained in later context.

$$\begin{array}{@{}rcl@{}}  &S^{c}_{1to1}=\{130, \underline{\mathbf{199}}, \underline{277}, 346\},\\  &S^{e}_{1to1}=\{\underline{132}, 182, 234\},\\  &S^{e}_{2to1}=\{263, \underline{363}, 467\}. \end{array} $$

We first deal with $S^{c}_{1to1}$. From the calculations in Table 1, we get that values 130, 199, and 346 satisfy ∣(199−130)−*a*_*S*_+18∣=0 and ∣(346−199)−*a*_*E*_−18∣=0, where *a*_*S*_=87 and *a*_*E*_=129 are the masses of serine and glutamine, respectively. Then we infer that the ion having *m*/*z* value of 199 (in boldface above) is a charge +1 fragment ion having a molecular of water loss, and score ${ss}_{199} = S^{aa}_{SL-HCD}$, where $S^{aa}_{SL-HCD}$ is the amino acid difference score calculated on a HCD spectra library. In addition, we get that values 199 and 277 (underlined above) satisfy ∣492+2*m*_*H*_−(199+277)−18∣=0. Then we infer that these two ions are complimentary ions in charge state +1. *s* *s*_199_ is updated to be ${ss}_{199} = S^{aa}_{SL-HCD} + S^{comp}_{SL-HCD}$, and ${ss}_{277} = S^{comp}_{SL-HCD}$, where $S^{comp}_{SL-HCD}$ is ion complementarity score calculated on a HCD spectra library.

We now deal with $S^{e}_{1to1}$ and $S^{e}_{2to1}$. Values 132 and 363 (underlined above) satisfy ∣492+3*m*_*H*_−(132+363)∣=0. Then we infer that these two ions are complimentary ions, and the ion having *m*/*z* value of 182 is in charge state +2 (the ion at the same position as ion 363 in $S^{e}_{1to1}$). Ion complementarity score calculated on a ETD spectra library, $S^{comp}_{SL-ETD}$, is assigned to both ions.

At this point, no more ions can be found satisfying the relationships described in Table 1. Therefore, the final merged spectrum *S*_*m*_ is $S_{m}=\left \{\left (132, S^{comp}_{SL-ETD}\right),\right.\left.\! \left (363, S^{comp}_{SL-ETD}\right), \left (\!199, S^{aa}_{SL-HCD} +^{comp}_{SL-HCD}\right), \left (277, S^{comp}_{SL-HCD}\right)\!\right \}$.

### De novo sequencing modification

In the sequencing part, we first extend the peptide tags and re-rank them. The previously used tags are partial sequences consisting of three amino acids. If two tags *t*_*i*_,*t*_*j*_∈*T* (*T* is the tag set) have two successive amino acids overlap and *m*/*z* values associated with the two tags have overlap, then a new tag *t*_*ij*_ consisting of four amino acids is generated and added into *T*. Let us say *t*_*i*_=*T* *A* *G* and *t*_*j*_=*A* *G* *T* where the overlapped amino acids are *A* *G*,*t*_*i*_ is generated by peaks *I*_1_,*I*_2_,*I*_3_,*I*_4_, and *t*_*j*_ is generated by peaks *J*_1_,*J*_2_,*J*_3_,*J*_4_. *I*_*x*_ and *J*_*x*_ are also used to represent their *m*/*z* values, where *x*=1,2,3,4. If ∀|*I*_*x*_−*J*_*x*−1_|≤*θ*, where *x*=2,3,4, then a new tag *t*_*ij*_=*T* *A* *G* *T* is generated, and *T*⇐*t*_*ij*_∪*T*. If there is another tag *t*_*p*_=*G* *T* *A* where *t*_*p*_∈*T*, and the *m*/*z* of the ions generating *t*_*ij*_ and *t*_*p*_ satisfying the above relationship, a new tag *t*_*ijp*_=*T* *A* *G* *T* *A* is generated, and *T*⇐*t*_*ijp*_∪*T*. This process continues until no more new tags can be generated. For each tag *t*∈*T* with length *l*_*t*_, it is generated by *l*_*t*_+1 peaks in an experimental spectrum. Typically, amino acids in the middle of a tag, for example, the *AT* in *t*_*ij*_, tend to be more reliable than the amino acids in the ends, for example,the two *T*s in *t*_*ij*_. Since each peak has a score calculated from above subsection, the score of *t*, denoted as *s*_*t*_, is a sum of the *l*_*t*_+1 peaks with proper weights to all peaks. *s*_*t*_ is calculated using Eq. 1. Here, *s* *s*_*l*_ is the score of *l* ^*th*^ peak in tag *t*. (1+0.1×*m* *i* *n*{*l*,*l*_*t*_−*l*}) is the weight assigned to the *l* ^*th*^ peak. With this calculation, peaks in the two ends have lower weights and peaks in the middle parts have higher weights.

1 $$ s_{t} = \frac{1}{l_{t}+1} \sum^{l_{t} + 1}_{l=1} {ss}_{l}\left(1+0.1 \times min\lbrace l, l_{t}-l\rbrace\right)  $$

All the tags *t*∈*T* are then ranked according to *s*_*t*_, and *Sel* tags with highest ranking are selected for the following sequencing. The set of the selected tags is denoted as *TS*.

Having a tag *t* *s*∈*T* *S*, the graph model with multiple types of edges are applied to find candidate peptide sequences. Since each peak has a score, the algorithm searches from the highest scored peak to extend paths, and a threshold is used here to stop the searching. Here, when *K* paths are successfully found, the searching stops. Here *K* is a user defined threshold.

### Candidate ranking

Each candidate peptide *P*_*cp*_ is generated by finding a proper path in the graph model. Since each vertex on the path represents a peak in the merged spectrum, the score of *P*_*cp*_, denoted as *c* *s*_*cp*_, is defined as the peaks’ score sum of all the peaks on the path generating *P*_*cp*_. When all candidate peptides are generated, we rank them with their score *P*_*cp*_, and output highest *C* candidates and their scores. Here, *C* can be defined by users.

## Results and discussion

In this section, we use two spectra libraries, one containing HCD spectra and the other containing ETD spectra, as training datasets to calculate significant scores introduced above. Two pairs of HCD and ETD spectral datasets are used to test the performance of the proposed de novo sequencing method. The comparison to our previous method and results analysis are given as well.

### Spectra libraries and MS/MS data

In this study, two spectra libraries consisting of annotated HCD and ETD spectra respectively are used. The first library of HCD spectra is from The National Institute of Science and Technology (NIST) website (chemdata.nist.gov). NIST has built MS/MS spectra libraries for several model organisms and made them publicly available [20]. We use the human peptide spectral library (built date Nov 24, 2014) containing 183,140 spectra measured with Orbitrap-HCD. The other library is a peptide library of over 100,000 synthetic, unmodified peptides and their phosphorylated counterparts, and they were analysed by both HCD and ETD fragmentation of MS/MS [21]. Among them, the ETD spectra of unmodified peptides are used in this study. The annotated peptide associated with each spectrum in these libraries was used as the correct sequence of such spectrum.

Experimental MS/MS spectra used here are similar as the ones in our previous study [11]. Two pairs of HCD and ETD spectral datasets, SCX _*HCD*_decon and SCX _*ETD*_decon, plus SCX _*HCD*_*no*_decon and SCX _*ETD*_*no*_decon, are used here. These pairs of datasets are from the same research paper [22]. The latter dataset pair (labeled with “ _*no*_decon”) contains raw data without deconvolution of spectra while the other pair contains spectra with deconvolution [11]. The original datasets contain spectra analysed by CID, HCD, and ETD fragmentation. Each spectrum has a sequence associated with it. The HCD and ETD spectra pairs having the same peptide sequences were selected first, and those pairs that can be successfully sequenced using only single spectrum separately are filtered out. Methods used in this filtration are NovoHCD [9] and NovoGMET [10], respectively. The reason for this filtration is that the focus of de novo sequencing using multiple spectra is for those ones that can not be sequenced by using just one spectrum. The number of spectra, the charges of spectra, and the number of selected pairs of spectra for experiment are summarized in Table 3.

**Table 3:** Number of spectra and charges in each dataset used in the experiments

Dataset	Number of	Charge of	Number of
	total spectra	spectra	selected pair
SCX _*H* *C* *D*_decon	1952	+2 to +6	161
SCX _*E* *T* *D*_decon	612		
SCX _*H* *C* *D*_*n* *o*_decon	2557	+2 to +5	249
SCX _*E* *T* *D*_*n* *o*_decon	1298		

### Parameters

There are several parameters usesd in the proposed method, and the values applied in the experiments are listed in Table 4. *θ*, number of tags generated for each experimental spectrum, and number of output candiates are set according to our previous study and experiments [11]. The number of tags is chosen to be 10 because the tags ranked lower than the top 10 tags are most likely to be wrong tags according to our previous study [9].

**Table 4:** Parameters used in the experiments

Parameter	Role in the method	Value
Threshold *θ*	Peak selection in spectra merging	0.01Da
Number of tags	De novo sequencing	10 per experimental spectrum
Stop threshold *K*	Path extending in sequencing	10 in each partial segment
Output number *C*	Candidate output	3 per spectra pair

### Score calculation

When using spectra libraries to calculate significant scores of the selection criteria, we investigate the score variation on different peaks in a spectrum. The results show that for the ion complementarity score on HCD spectra, the peak pairs in the middle of a spectrum tend to generate lower significant scores than the pairs in the two ends of a spectrum. Therefore, for a spectrum *S* *L*_*k*_ in NIST-HCD library, we divide the peaks on *S* *L*_*k*_ into four parts evenly according to the highest *m*/*z* valued peak. Then, $S^{comp}_{SL}$ calculated using peak pairs in the middle two parts and the end two parts, denoted as $S^{compM}_{SL}$ and $S^{compE}_{SL}$ respectively, are shown in Table 5.

**Table 5:** Significant score calculated using spectra libraries

Score	Calculation on NIST-HCD	Calculation on SynthETD
$S^{compM}_{SL}$	0.79	0.42
$S^{compE}_{SL}$	0.92	
$S^{aa}_{SL}$	0.61	0.66

From Table 5 one can see the scores of peak pairs on different positions on a spectrum are distinguishable, and it is necessary to use two scores to represent them. We then investigate the same score on SynthETD library. However, the score variation is slight on it (0.38 to 0.44). Therefore, we use just one $S^{comp}_{SL}$ score for ETD spectra.

For the score $S^{aa}_{SL}$, preliminary results show that this change is not significant as well (0.54 to 0.62 on NIST-HCD library and 0.61 to 0.68 on SynthETD library). In addition, considering that there will be 400 slightly different scores if we distinguish the 20 amino acids, we just use one score, $S^{aa}_{SL}$, to describe the significance of the amino acids differences. The values of $S^{aa}_{SL}$ calculated on the two spectra libraries are shown in Table 5.

### De novo sequencing performance

We first investigate the full length sequencing accuracy of the proposed method and our previous method. Our previous method output the three highest ranked peptide candidates for each spectra pair, and if any one of them matches the correct sequence, we say that this pair of spectra are correctly sequenced. Here, the same criterion is applied to the proposed method. The accuracy comparison of the proposed method and previous method using two pair of HCD and ETD datasets is shown in Table 6. Results on the previous method are from the orignal research paper presented them [11].

**Table 6:** Full length peptide sequencing accuracy comparison on two HCD and ETD dataset pairs

Dataset	Number of	Accuracy of	Accuracy of the
	spectra	previous	proposed
	pairs	method	method
SCX _*HCD*_decon	161	83.53%	86.96%
and SCX _*ETD*_decon			
SCX _*HCD*_*no*_decon	249	94.78%	95.16%
and SCX _*ETD*_*no*_decon			

One can see from the results that the proposed method has similar accuracy compared to the previous method. This indicates that with the use of longer peptide tags and stop criterion (threshold *K*), the proposed method maintains the performance without any drop of accuracy. The proposed method does has a slight accuracy increase on these two pairs of datasets. After further investigating the results, it shows that the increase is because of the new ranking score of candidate peptides. Correct sequences are ranked higher (within top three) using the new ranking scores for those newly sequenced spectra, compared to the previous method, which are ranked out of the top three.

We then further analyse the rankings of candidate peptides sequenced from the proposed method since that it is a major difference between the proposed method and the previous method. One situation in the previous method is that often, several top ranked ones have very similar ranking scores. (We omit the detials of the ranking scheme here to aviod reduandency, and details can be seen in [11]). The correct sequence may not always be the highest scored one (ranked as first), but second or third ranked ones who has similar ranking scores as the highest one. So the previous method outputs all 3 highest ranked candidates. In this study, we would like to improve the ranking scheme with the significant scores calculated from spectra libraries. In the following, in order to show the contribution of the new ranking scheme, we compare the accuracy differences of the following three cases: output only the highest ranked one, the top two ranked ones, and the top three ranked ones. Results of the previous and proposed method on two different dataset pairs are shown in Tables 7 and 8.

**Table 7:** Accuracy comparison on SCX _*HCD*_decon and SCX _*ETD*_decon dataset pair with different output

Method	Output first	Output first and second	Output top three
Previous	65.22%	72.05%	83.85%
Proposed	76.40%	80.12%	86.96%

**Table 8:** Accuracy comparison on SCX _*HCD*_*no*_decon and SCX _*ETD*_*no*_decon dataset pair with different output

Method	Output first	Output first and second	Output top three
Previous	80.43%	84.04%	94.78%
Proposed	82.71%	87.94%	95.16%

From these figures one can see that the new ranking scheme has better performance than the one used in the previous method. With this new approach, more of the highest ranked candidates are the correct sequences, with an increase up to 11% compared to the previous method, if only outputting the first ranked candidates.

Finally, the computational time of the proposed method and our previous method is compared in Table 9. Both algorithms were written using MATLAB (2010b) and run on a PC with a 3.07 GHz quad-core CPU and MS Windows 7 operating system. Since the proposed method uses longer tags and limits the number of paths in the graph model, it uses less computational time for calculation compared to the previous method. The time saving is about 25 and 40% on the two pair of HCD and ETD datasets.

**Table 9:** Computational time comparison on two HCD and ETD dataset pairs

Dataset	Number	Time (sec.) per	Time (sec.) per
	of spectra	pair using	pair using
	pairs	previous method	proposed method
SCX _*HCD*_decon	161	3.97	2.16
and SCX _*ETD*_decon			
SCX _*HCD*_*no*_decon	294	2.35	1.79
and SCX _*ETD*_*no*_decon			

## Conclusions

In this paper, an improved de novo sequencing method assisted with spectra library for HCD and ETD spectra pairs is proposed. It is a development of our previous proposed method for the same problem [11]. The proposed method uses spectra libraries as training datasets and introduces significant scores to the spectra merging criteria of the previous method. In addition, the use of tags is improved; the original length-three tags (three amino acids long) are extended to be longer tags in this method.

Two spectra libraries, one of HCD and the other of ETD spectra, were used to generate signigicant scores. To investigate the performance of the proposed method, two pairs of HCD and ETD spectral datasets were used for test and compared with our previous method. When outputting top three ranked candidates, the proposed method has a slight increase in terms of sequencing accuracy compared to the previous method. But the accuracy differs significantly when outputting only top one ranked candidates. In the latter case, the proposed method achieved higher accuracy up to 11% increase, compared to the previous method. In addition, with longer peptide tags used, the proposed method uses less computational time than the previous method, with a time saving up to 25 and 40% on the two pair of experimental spectral datasets. To summarize the advantages of this proposed method, it achieves better de novo sequencing accuracy with higher ranked correct sequences and less computational time.

In future, we would like to evaluate the proposed method on more MS/MS datasets, and further study the spectra library to integrate more information to the de novo sequencing methods for enhanced performance.
